# Expression profile of innate immune receptors, NLRs and AIM2, in human colorectal cancer: correlation with cancer stages and inflammasome components

**DOI:** 10.18632/oncotarget.5587

**Published:** 2015-09-10

**Authors:** Rongrong Liu, Agnieszka D. Truax, Liang Chen, Peizhen Hu, Zengshan Li, Jun Chen, Chaojun Song, Lihua Chen, Jenny Pan-Yun Ting

**Affiliations:** ^1^ Department of Immunology, School of Basic Medicine, Fourth Military Medical University, Xi'an, China; ^2^ The Lineberger Comprehensive Cancer Center, University of North Carolina at Chapel Hill, Chapel Hill, NC, USA; ^3^ Department of Pathology, Xijing Hospital, Fourth Military Medical University, Xi'an, China; ^4^ Department of Encephalopathy, Traditional Chinese Medicine Hospital of Shan Xi Province, Xi'an, China; ^5^ Departments of Genetics, Microbiology-Immunology, University of North Carolina at Chapel Hill, Chapel Hill, NC, USA

**Keywords:** NLRs, AIM2, FFPE, colorectal cancer, inflammasome

## Abstract

NLRs (nucleotide-binding domain leucine-rich repeat proteins or NOD-like receptors) are regulators of inflammation and immunity. A subgroup of NLRs and the innate immune receptor, AIM2 (absent-in-melanoma 2), can induce the assembly of a large caspase-1 activating complex called the inflammasome. Other NLRs regulate key signaling pathways such as NF-kB and MAPK. Since inflammation is a central component of colorectal cancer (CRC), this work was undertaken to analyze NLR and AIM2 expression in human CRC by combining bioinformatics analysis and experimental verification using clinical tissue samples. Additional experiments analyzed the association of (i) gene expression and cancer staging, and (ii) gene expression among inflammasome components.

Ten public CRC datasets from the Oncomine^®^ Platform were analyzed. Genes analyzed include NLRP1, NLRP3, NLRP6, NLRP12, NLRC3, NLRC4, NLRC5, NOD1, NOD2 and AIM2. Additionally, forty case-matched cancer samples and adjacent healthy control tissues isolated from a cohort of Chinese CRC patients were profiled.

Three patterns of gene expression in CRC are shown. The expression of NLRC3, a checkpoint of inflammation, and the inflammasome components NLRP1, NLRP3, NLRC4 and AIM2 were reduced in CRC. NOD1 and NOD2 expression was increased in CRC, while NLRC5, NLRP6 and NLRP12 showed little difference compared to controls. Reduced expression of NLRC3 in CRC was verified in all available databases analyzed and confirmed with our patient cohort. Furthermore, the extent of NLRC3 and AIM2 gene reduction was correlated with cancer progression. This report reveals the potential value of NLR and AIM2 genes as biomarkers of CRC and cancer progression.

## INTRODUCTION

Inflammation is one of the hallmarks of cancer. Whereas acute inflammation protects against infectious pathogens, chronic inflammation is associated with DNA and tissue damage, including genetic and epigenetic changes leading to cancer. A common feature of gastrointestinal inflammatory diseases that leads to colorectal cancer (CRC) is the exaggerated production of cytokines by resident innate immune cells. These proinflammatory mediators stimulate the secretion of molecules that damage the intestinal epithelium and further amplify the response by recruiting and activating additional immune cells [1].

In the human intestine, trillions of bacteria interact with the host immune system in a complex balance between immune activation and tolerance. Pattern recognition receptors (PRRs) are essential components of the host immune system and significantly contribute to cancer pathobiology. There are at least four families of PRRs that have been implicated in tumorigenesis, including the toll-like receptors (TLRs), the NOD-like receptors (NLR), C-type lectin receptors (CLRs), and RIG-I-like receptors (RLRs). To date, the majority of studies assessing PRR signaling in cancer have focused on members of the TLR family. However, new and emerging findings in murine models have revealed a significant role for members of the NLR family in contributing to a variety of hallmarks associated with cancer including inflammation, cell death, tumor growth, angiogenesis, invasion, and metastasis [2].

There are 22 NLR members in humans and 34 members in mice [2]. A functional sub-group of NLRs has been identified as driving formation of a multi-protein complex termed the inflammasome [3]. The inflammasome is composed of an NLR that recognizes either a specific repertoire of pathogen associated molecular pattern (PAMP) or autologous damage associated molecular pattern (DAMP) molecules, the adaptor protein ASC, and pro-caspase-1. These subunits continue to multiplex, ultimately resulting in the maturation and activation of caspase-1, which subsequently drives the cleavage and activation of IL-1 and IL-18 [3]. The complex roles of inflammasome activation in cancer development or therapy have received much attention. In addition to NLR-based inflammasomes, AIM2, a member of the AIM2-like receptors (ALRs) that contain pyrin and hin domains, is crucial for inflamamsome activation induced by double-stranded DNA. AIM2 reduced Akt activation and tumor burden in colorectal cancer models, while an Akt inhibitor reduced tumor load in Aim2^−/−^ mice [4]. In the DSS colitis model and DSS-AOM colitis associated colon cancer murine models, several inflammasomes including NLRP3, NLRP6 and NLRC4 have been found to protect against dysplasia and hyperplasia in the colon.

CRC is one of the major causes of morbidity and mortality in developed countries. It is ranked the third most common cancer among males and females in the United States according to Cancer Statistics for 2015. Recent data have shown that the incidence rates of CRC has rapidly increased in China [5]. As a majority of patients are still diagnosed at a late stage, biomarkers that may allow early diagnosis are crucial [5]. Inflammatory molecules represent a possible source of CRC biomarkers, because the molecular pathobiology of CRC implicates pro-inflammatory conditions as promoting factors in the malignant progression of the tumor, invasion, and metastasis. It is well known that patients with inflammatory bowel disease such as Crohn's disease and ulcerative colitis are at higher risk of CRC, thus providing a link between inflammation and CRC [6]. Even among CRC that are linked to genetic mutations, contribution from inflammation to tumor development has been implicated, as shown by decreased CRC mortality with the regular use of nonsteroidal anti-inflammatory drugs (NSAIDs). These data strongly support a pro-tumorigenic role of inflammation in colon cancer [6].

Thus far, there are multiple studies of NLRs and AIM2 in animal CRC models [4, 7, 8], but there is a paucity of papers that have analyzed these genes in human CRC. In this report, we performed an extensive analysis of NLRs and AIM2 in human CRC by combining the bioinformatics analysis of ten independent, publically available databases with experimental validation using tissue samples from CRC patients. The study shows that NLRC3, an NLR protein that has been reported to negatively regulate inflammatory cytokines and NF-kB activity [9, 10], was reduced in CRC relative to controls in all public databases analyzed and this finding was substantiated in primary CRC patient samples. This indicates the robustness of data regarding reduced NLRC3 expression in CRC. In addition, the expression of inflammasome NLRs and AIM2 in CRC was significantly lower than normal controls in the TCGA database, suggesting that these genes could be important for maintaining tissue homeostasis against tumorigenesis. An analysis of primary patient samples showed that the expression of a major inflammasome NLR, *NLRP3,* was reduced in primary CRC samples. Additional correlative analyses of gene expression with cancer stages, tumor anatomic locations and other components within the inflammasome pathway were conducted. Based on this broad analysis, NLRC3 and several inflammasome genes may represent valuable candidate biomarkers for CRC screening.

## RESULTS

### Expression levels of NLRs and AIM2 in colorectal cancer culled from the Oncomine^®^ Platform

The Oncomine^®^ Platform (http://www.oncomine.org) is an online collection of microarrays where data are analyzed and standardized uniformly thus allowing for an easy comparison across studies. We initially analyzed the expression levels of NLRP1, NLRP3, NLRC3, NLRC4, NLRC5, NLRP6, NLRP12, NOD1, NOD2 and AIM2 using the Oncomine® Platform CRC database to compare healthy versus cancer tissues. Among the genes analyzed, all except for NLRC3 have at least one paper supporting their function in inflammasome activation, leading to caspase-1 activation to process pro-IL1*-*β and pro-IL-18 [11]. However, several of these genes have been reported to display other functions, and some cases the other functions are the more consistently observed ones. For example, the main function of NLRC5 is regulating class I Major Histocompatibility Complex (MHC) genes [12], while NOD1/NOD2 proteins are primarily required for the positive regulation of NF-κB and MAPK [13]. NLRP6 and NLRP12 have been found to attenuate NF-kB and MAPK in models of colitis and CRC [14–17], NLRC3 has been shown to act as a negative regulator of TLR and DNA-induced cytokine production [9, 10].

**Figure 1:**
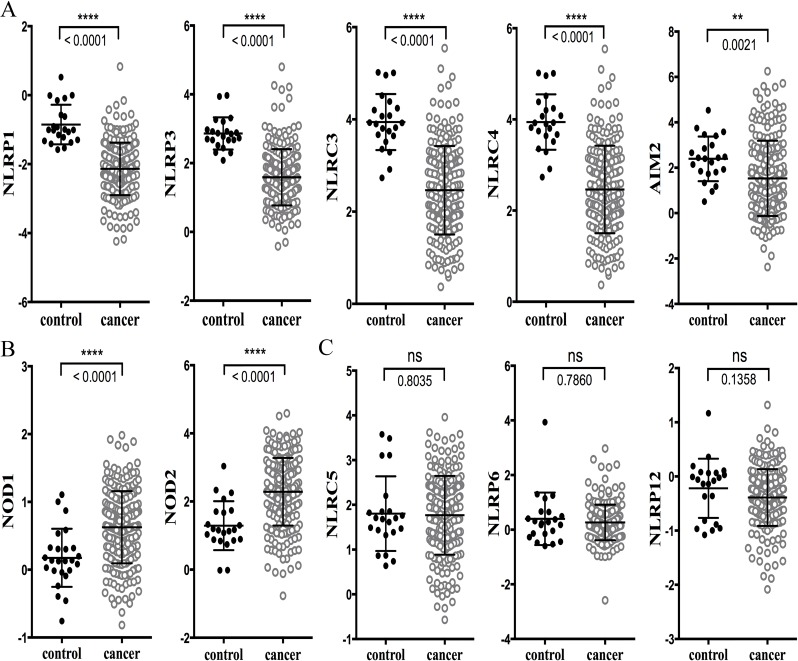
Analysis of microarray gene expression data for NLRs and AIM2 in the TCGA database The raw data was exported from the TCGA CRC database, with representations of 22 samples from healthy control and 215 samples from CRC. **A.** Expression levels of NLRP1, NLRP3, NLRC3, NLRC4 and AIM2 were significantly decreased in human colorectal cancer; **B.** NOD1 and NOD2 had significantly higher levels of expression in CRC; **C.** NLRC5, NLRP6 and NLRP12 expression was slightly reduced in CRC but statistical significance was not reached. Data are expressed as mean ± SEM. Log2 median-centered ratio expression. Unpaired Mann-Whitney test was used to evaluate the significance of differential mRNA expression levels of candidate genes. ** indicates *P* < 0.01; **** indicates *P* < 0.0001; ns, no statistical difference.

Ten databases are listed in Materials and Methods. These databases have different number of patients and contain different clinical information. We choose to analyze the Cancer Genome Atlas (TCGA) which contained the most comprehensive colon database as a starting point for our analysis [18]. Analysis of the TCGA database showed that the expression of analyzed genes can be grouped into three patterns. In the first one, the expression levels of NLRP1, NLRP3, NLRC3, NLRC4 and AIM2 in CRC were significantly lowered in CRC than in healthy controls (Figure 1A, data summarized in Table 1). In the second group, an opposite pattern was observed with NOD1 and NOD2 (Figure 1B and Table 1), where these genes were significantly higher in CRC than controls. In a final group, NLRC5, NLRP6 and NLRP12 expression was not statistically different from controls (Figure 1C and Table 1). Table 1 also shows gene expression comparisons between CRC and controls in colon adenocarcinoma, rectal adenocarcinoma and cecum adenocarcinoma. No distinct differences were observed among these different cancers, indicating that levels and patterns of expression are comparable amongst various colorectal cancers at different anatomic locations.

**Table 1:** Statistical analysis of NLRs and AIM2 expression in CRCs from different anatomical localizations

	Colon Adenocarcinoma VS. Normal		Rectal Adenocarcinoma VS. Normal		Cecum Adenocarcinoma VS. Normal	
	Fold Change	P-value	Fold Change	P-value	Fold Change	P-value
**NLRX1**	−1.63	1.85E-18	−1.73	1.93E-15	−1.45	3.65E-05
**NLRC3**	−2.97	3.63E-11	−3.49	7.89E-13	−2.45	8.96E-08
**NLRP3**	−2.50	1.46E-14	−2.81	5.78E-17	−2.34	5.20E-10
**NLRP1**	−2.51	1.10E-11	−2.58	6.45E-12	−2.19	2.46E-06
**NLRC4**	−2.64	1.19E-11	−3.51	5.20E-15	−3.02	1.60E-07
**AIM2**	−1.88	5.20E-04	−2.09	4.70E-04	−1.45	1.02E-01
**NLRP12**	−1.17	0.039	−1.19	0.056	−1.23	0.036
**NLRP6**	−1.08	0.292	−1.1	0.281	−1.14	0.216
**NLRC5**	1.01	0.535	−1.16	0.144	−1.01	0.458
**NOD1**	1.52	3.44E-08	1.5	1.78E-07	1.4	2.61E-04
**NOD2**	2.14	6.16E-08	1.86	9.41E-06	1.68	8.89E-04

TCGA database in the Oncomine® Platform was analyzed. Fold change was calculated by taking the average of 102 colon adenocarcinoma, 60 rectal adenocarcinoma or 24 cecum adenocarcinoma samples compared with 22 normal controls. Results are expressed as means between the controls and CRC groups in a differential expression analysis using the Oncomine® Platform.

### Expression levels of NLRs and AIM2 in various stages of colorectal cancer progression

**Figure 2:**
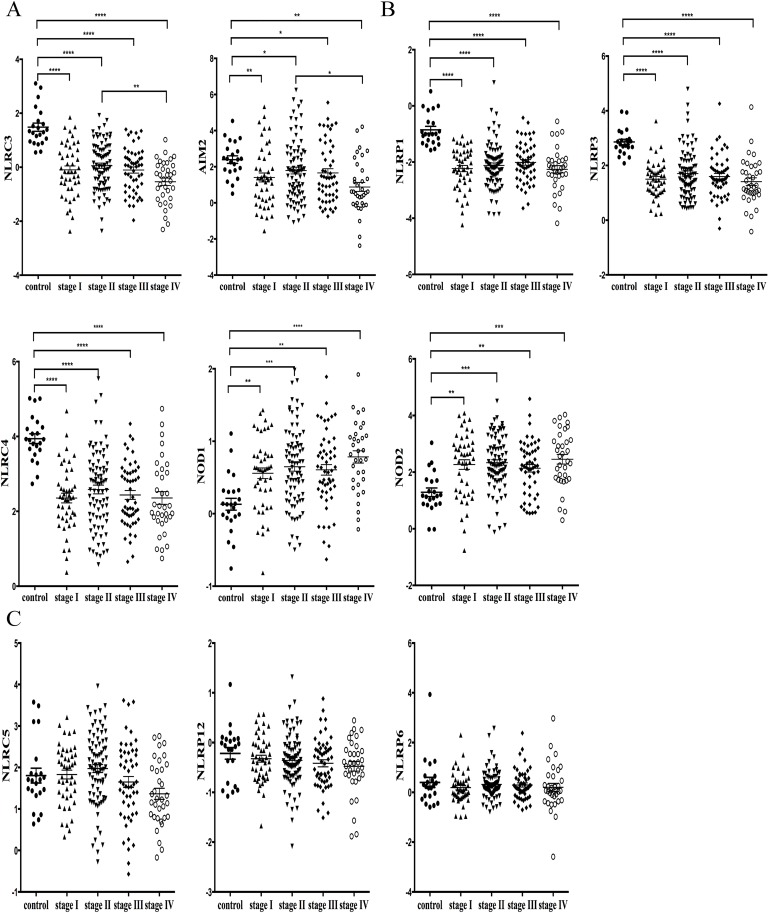
Correlation analysis comparing expression levels of NLRs and AIM2 among different stages of CRC progression TNM stage information was exported from the TCGA colon database. **A.** NLRC3 and AIM2 showed statistically different levels of expression between healthy control and various CRC stages of cancer progression, where stage IV exhibited significantly lower expression of these two genes than stages 1-3. **B.** NLRP1, NLRP3, NLRC4, NOD1 and NOD2 exhibited statistically different levels of expression between healthy control and all four stages of CRC. **C**. Levels of NLRC5, NLRP12 and NLRP6 expression among control and cancer stages 1-4 were not significantly different. Data were presented as means ± SEM. *P < 0.05; **P < 0.01; **P < 0.001****P < 0.0001.

Next we analyzed the expression levels of NLRs and AIM2 at various stages of cancer progression in the TCGA database. CRC cases were staged according to the AJCC (American Joint Committee on Cancer) staging system, which also is known as the TNM (tumor-node-metastasis) system. In Stage I tumor has spread beyond the inner lining of the colon to the second and third layers and involves the inside wall of the colon. Stage II tumor is larger and extends through the muscular wall of the colon, while Stage III cancer has spread outside the colon to one or more lymph nodes. Finally, Stage IV cancer has metastasized to other areas of the body, such as the liver or lung [19]. Data presented in Figure 2A and 2B demonstrate that NLRC3, AIM2, NLRP1, NLRP3, NLRC4, NOD1 and NOD2 displayed statistically different levels of expression in healthy control *vs*. various CRC stages. Similar to Figure 1, the expression of NLRC3, AIM2, NLRP1, NLRP3 and NLRC4 was reduced in all stages of CRC relative to healthy controls, while the expression of NOD1 and NOD2 were higher in CRC compared to controls. NLRC3 and AIM2 (Figure 2A) were the only two genes that showed statistical difference between stage IV CRC and stages I-III CRC, wherein their expression in stage IV CRC was significantly lower than the other stages of CRC. These results suggest that NLRC3 and AIM2 may be useful as biomarkers of cancer progression. By contrast, levels of NLRC5, NLRP12 and NLRP6 did not differ between stages of cancer progression (Figure 2C).

In sum, these results obtained from the TCGA database indicate that across tumor stages, NLRP1, NLRP3, NLRC3, NLRC4 and AIM2 expression in CRC tissues was significantly less than healthy controls. In contrast, NOD1 and NOD2 expression was significantly higher than controls, while NLRP6, NLRP5 and NLRP12 expression was not statistically different from controls. Among the genes analyzed, AIM2 and NLRC3 expression was significantly reduced in stage IV CRC compared to the earlier tumor stages.

### Expression of NLRs and AIM2 in human colorectal cancer FFPE clinical samples from China

**Figure 3:**
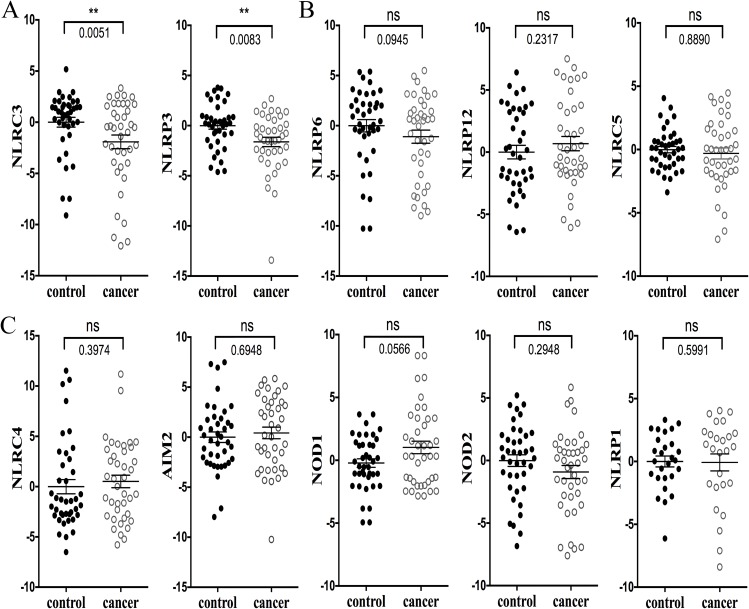
Expression of NLRs and AIM2 in human CRC FFPE samples (*n* = 40) The quantitative RT-PCR data were obtained by the comparative C_T_ method (2^−ΔΔCT^). **A.** The expression of NLRC3 and NLRP3 was significantly reduced in CRC. **B.** NLRP6, NLRP12 and NLRC5 expression was not significantly different between CRC and healthy controls. Results shown in **A.** and **B.** are congruent with the TCGA database presented in Figure 1. **C.** NLRC4, AIM2, NOD1, NOD2 and NLRP1 expression in FFPE CRC and adjacent normal controls were not significantly different. This data does not correlate with the TCGA database. The RT-PCR results are converted to a log2 scale. Data are expressed as mean ± SEM. Wilcoxon matched-pairs signed rank test was used to evaluate the significance of differential mRNA expression levels of candidate genes. *P < 0.05; **P < 0.01; **P < 0.001 ****P < 0.0001, and ns indicates the lack of a statistical difference. NLRP1 was only detected in 27 samples. Mann-Whitney unpaired test was used to perform statistical analysis on the GraphPad software.

Next, we compared forty freshly isolated CRC and forty adjacent normal FFPE tissues obtained from a Chinese patient cohort for the expression of NLRs and AIM2 by Q-RT-PCR. As shown in Figure 3A levels of NLRC3 and NLRP3 were significantly reduced in CRC while levels of NLRP6, NLRP12 and NLRC5 (Figure 3B) were not significantly different between CRC and controls. These data are in agreement with the TCGA database presented in Figure 1. However, expression levels of NLRC4, AIM2, NOD1, NOD2 and NLRP1 (Figure 3C) in FFPE CRC and adjacent normal controls were not significantly different and did not correlate with the TCGA database presented in Figure 1. There are several caveats that could account for the difference between the TCGA database and our patient cohort. Some of the genes were detected at a very low level. For example, 13 out of 40 (32.5%) FPPE human samples had undetectable levels of NLRP1, and this low level of expression made the analysis definitely less reliable. Another caveat is that the experimental FPPE samples represent a small patient cohort. Finally, while the FFPE samples were obtained from a Chinese cohort, the ten Oncomine® Platform databases were collected from patients outside of China, which may reveal groups with different genetic variations. As an illustration, while NOD2 has been strongly associated with Crohns’ disease in patients from the Western world, this has not been observed among Asian patients [20–22]. This observation is in agreement with our own analyses where NOD2 levels of expression were statistically higher in CRC based on multiple databases (Figure 1B), but yet it did not reached statistical significance in FFPE samples from the Chinese cohort (Figure 3C). In summary, the bioinformatics analyses of the TCGA database and the experimental analysis of clinical samples from China both showed reduced expression of NLRC3 and NLRP3 in CRC samples.

### The expression of inflammasome genes and correlation with NLRs and AIM2

**Figure 4:**
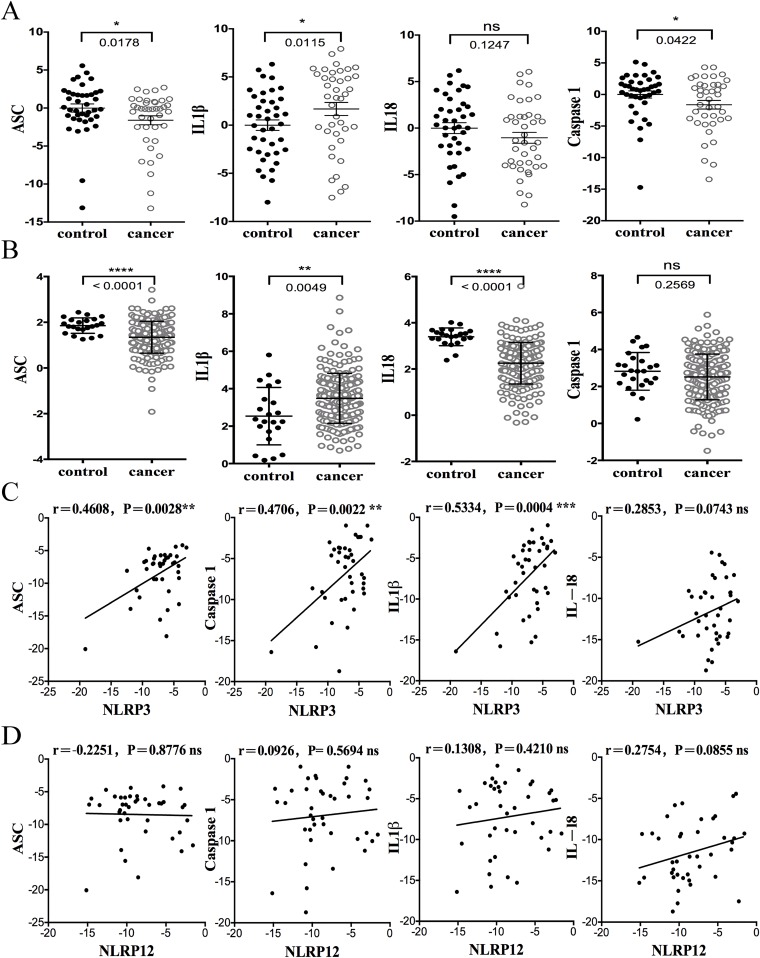
The expression of inflammasome genes in human CRC FFPE samples and correlation with NLRs and AIM2 **A.** The expression of ASC, caspase 1, IL-1β and IL-18 in human CRC samples from our Chinese cohort. **B.** The expression of ASC, caspase 1, IL-1β and IL-18 in the TCGA CRC database. **C.** The mRNA expression level of NLRP3 in human CRC samples was positively correlated with ASC, Caspase1, IL1β and IL18. The quantitative real-time PCR is graphed using 2-ΔΔCT for correlation analysis and subsequently converted to a log2 scale. The r and p values are indicated in the graphs. Linear regression and association analyses were generated by GraphPad. **D.** NLRP12 did not show significant correlation with inflammasome components.

One of the most important functions of the NLR and ALR family is to serve as central components of an inflammasome. Inflammasome is composed of an NLR or AIM2, which serves as the sensor or receptor that recognizes PAMPs or DAMPs, the adaptor protein ASC and pro-caspase 1 Inflammasome activation depends on the expression of the multiple inflammasome protein components in the same cell. ASC and caspase-1 are found in many tissues and cell types and have been shown to protect against colitis-associated CRC in mice [23]. IL-1β mRNA expression is primarily found in myeloid cells, although it is also expressed by other cell types and its expression is enhanced in a number of cancers including lung, colon, melanoma, and breast. Functionally, it is a potent pro-inflammatory cytokine associated with tumor growth and angiogenesis [24]. Since the analysis of Oncomine® Platform databases and FFPE samples consistently showed that NLRP3 was reduced in CRC, we analyzed the corollary expression of NLRP3 with the other inflammasome components in CRC and control samples. Figure 4A shows that mRNA expression of two inflammasome components, ASC and caspase1, and the downstream substrates of caspase-1, IL-1β and IL-18, in human FFPE CRC samples. Levels of ASC and caspase-1 were significantly lower in human CRC samples relative to their healthy adjacent colon tissues. Consistent with the literature, the level of IL-1β was statistically elevated in these CRC samples. Pro-IL-18 is also cleaved by caspase-1 into its mature form and has been reported to play both beneficial and detrimental roles in the progression of cancer [25]. However our data show that the level of IL-18 expression was not statistically different between CRC and adjacent controls from this cohort. By contrast, IL-18 was significantly reduced in the TCGA database, as was ASC (Figure 4B). IL-1β expression in CRC was increased in the TCGA database, but caspase-1 was not different. The difference between our patient cohort and the TCGA database parallels the confounding literature regarding IL-18 and cancer. In humans, an increase in IL-18 has been correlated with various types of cancer including ovarian carcinoma, head and neck squamous carcinoma, breast cancer, and others [26, 27]. However, in models of colitis-associated colorectal cancer IL-18 cytokine add-back showed that IL-18 protected against tumor [28]. The administration of IL-18 also induced anti-tumor immunity in mice bearing B16 melanoma tumors expressing B7-1 (CD80) [29]. Hence, the roles of IL-18 in cancer and cancer models are complex and may vary depending on the types of tumors and the companion therapy used to treat the tumor.

Inflammasome-forming NLRs have been found to significantly regulate the tumor microenvironment by modulating cytokine production [3], and the TCGA database and our patient cohort showed reduced NLRP3 expression. Thus we examined the level of NLRP3 expression in primary CRC tissues and found that it was directly correlated with inflammasome components ASC, caspase-1, IL-1β and IL-18 (Figure 4C). By contrast, NLRP12 did not show a significant correlation with inflammasome components (Figure 4D). NLRP12 has been linked to inflammasome induction induced by a limited number of pathogens [30], but it additionally displayed functions beside inflammasome activation by reducing NF-kB and MAPK activation [15]. The lack of an associative expression between NLRP12 and the other inflammasome components may be attributed to these alternate functions of NLRP12.

### Analyses of NLRs and AIM2 expression in multiple databases

**Figure 5:**
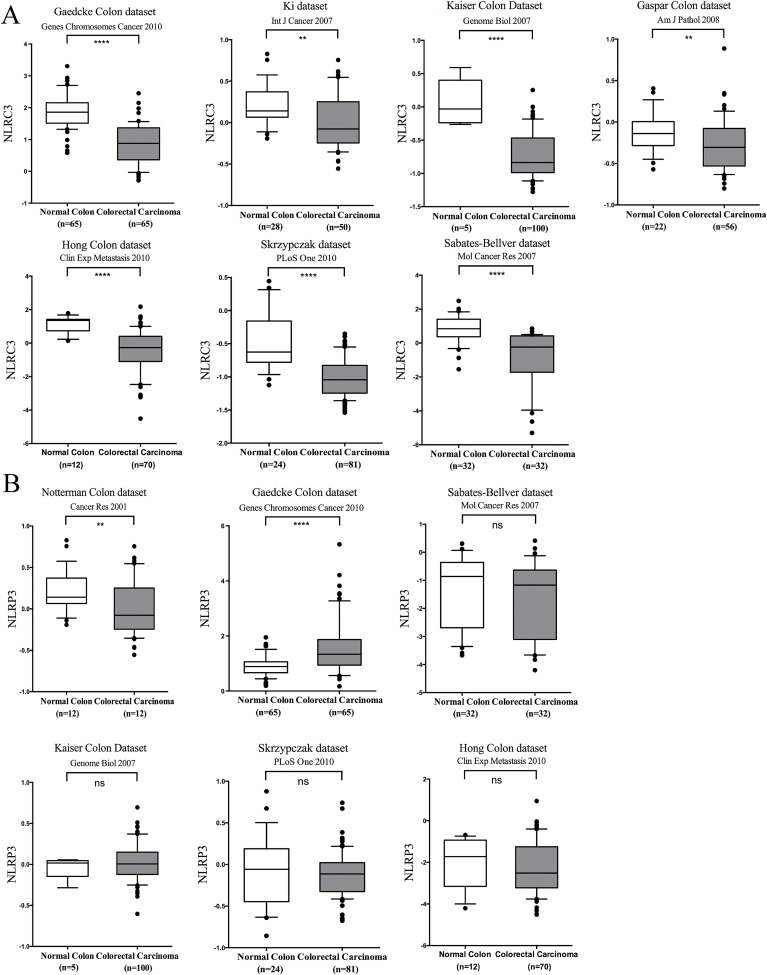
Analyses of NLRC3 and NLRP3 gene expression in multiple databases **A.** Expression of NLRC3 was significantly lower in CRC in all available databases; **B.** NLRP3 was significantly reduced in two out of six databases, but elevated in one of the databases and was not significantly different in the other 3 databases. Log2 median-centered ratio expression is present for 6 different datasets. Box plot were generated by GraphPad, which the whiskers are drawn down to the 10th percentile and up to the 90th. Points below and above the whiskers are drawn as individual dots. *P < 0.05; **P < 0.01; **P < 0.001 ****P < 0.0001. ns: no statistical difference.

To increase the power of this analysis, we assessed the expression of NLRs and AIM2 in nine additional databases within the Oncomine® Platform. Data from fewer than nine total databases are shown for each gene, because all of these genes are missing in one or more databases. In short, Figure 5A shows significantly lower expression of NLRC3 in all databases that contained this gene. This is in contrast with NLRP3 expression, which was significantly reduced in CRC in one, elevated in another and not significantly different from controls in three other databases (Figure 5B). In addition to NLRC3, which showed remarkable consistency, NOD1/NOD2 was higher in CRC in all except for one database (Supplementary Figure 1A and 1B), while NLRC5 expression in CRCs was not significantly different from controls in all-available databases (Supplementary Figure 2). By contrast, less consistency was observed for the other NLRs analyzed: NLRP1 was significantly reduced in two out of six databases (Supplementary Figure 3A); NLRC4 was significantly elevated in one but reduced in two databases (Supplementary Figure 3B); NLRP6 was significantly elevated in two but reduced in one out of six databases (Supplementary Figure 4A); NLRP12 was elevated in three out of six databases (Supplementary Figure 4B) and AIM2 was significantly reduced in two out of eight databases (Supplementary Figure 5). Variability across different databases could be due to variations in patient populations, patient selection criteria, patient numbers and sample collection. Conversely, the consistency of data collected for NLRC3, NOD1, NOD2 and NLRC5 gene expression among all of the databases underscores the remarkable reproducibility and robustness of findings pertaining to these four genes.

## DISCUSSION

Aberrant inflammation is considered both an emerging hallmark of tumorigenesis and an enabling characteristic of cancer. During the early stages of tumorigenesis, an inflammatory microenvironment serves as an enabling characteristic to activate diverse signaling pathways and drive the progression of pre-malignant and malignant lesions toward cancer. In later stages, cancer cells typically acquire a diverse repertoire of defense mechanisms that allow cells to both passively and actively evade immune surveillance and elimination [31]. This immune system subversion is an emerging hallmark of cancer and serves to remove the most effective barriers employed by the host to defend against neoplastic, late-stage tumor, and progression of micro-metastasis [31].

PRRs are essential mediators of the host immune response and have emerged as critical elements affecting multiple parts of tumor pathobiology, although their roles are likely complex as revealed by studies of murine models of cancer. NLR proteins are intracellular PRRs that sense microbial and non-microbial products. In CRC murine models, some NLRs exacerbated CRC, while others were thought to be essential in maintaining gut homeostasis. Recent characterization of NLRs has revealed several members that attenuate inflammation. Identifying the unique regulatory and signaling pathways modulated by these NLRs is an essential step towards the ultimate development of effective therapeutics targeting these proteins and the pathways they modulate. Characterizing unidentified ligands, dissecting the cell types that express these genes and defining their regulatory mechanisms should significantly improve our understanding of their contributions to CRC.

The expression of NLRs has not been previously analyzed in human CRC, but their gene expression in other cancers has been analyzed albeit on a limited scale. Mitchell *et al.* previously reported the association of genetic polymorphisms in NLRP3 and NLRP12 with gastric cancer in Chinese individuals [32]. Another study found that reduced AIM2 expression was closely associated with poor outcome for CRC patients [33]. A liver cancer study revealed reduced expression of NLRP3 inflammasome components in different stage of hepatocarcinogenesis (HCC) [34]. Finally, gene expression profiles of non-small cell lung cancer showed that NLRC4 was down regulated in lung cancer tissue [35].

Our data represent an extensive analysis of both Oncomine® Platform and clinical CRC tissues from our patient cohort. The TCGA database showed that expression levels of multiple inflammasome proteins including NLRP1, NLRP3, NLRC4 and AIM2 were significantly reduced in human CRC relative to healthy controls. Further analysis showed a strong corollary expression of NLRP3 with ASC, caspase-1, IL-1β and IL-18. These data support the findings in mouse CRC models, where deficiencies in several inflammasome genes have been shown to exacerbate disease outcome. In addition, we also showed reduced NLRP3 expression in CRC in both the TCGA database and our patient cohort. However the data were less consistent when other databases from the Oncomine® Platform were analyzed.

In contrast, the reduction of NLRC3 and elevation of NOD1 and NOD2 expression in CRC compared to control healthy tissue represent the most consistent findings across multiple databases. NLRC3 is a negative regulator of innate immune signaling induced by TLR via the TRAF6 molecule and by DNA via the STING pathway [9, 10]. It has not been previously evaluated in the context of any cancer model; however, its negative regulation of NF-κB signaling would be expected to attenuate tumorigenesis [15]. Indeed our data showed that NLRC3 expression was significantly reduced in all eight public databases analyzed, and was further reduced in samples obtained from our own patient cohort. NLRC3 also exhibited a lower level of expression in stage 4 CRC compared to stage 1-3 CRC, suggesting its potential role in cancer progression.

In contrast to NLRC3, the expression of NLRC5, NLRP6 and NLR12 did not show significant differences between controls and CRC samples. It is interesting that all three of these genes have been assigned multiple functions, including MHC gene regulation (for NLRC5), NF-kB and MAPK regulation (for all three), and inflammasome activation (for all three). The impact of their possible multiple functions and how each function might impact CRC remain to be determined.

Besides NLRC3, the most consistent finding among all databases analyzed is the elevated expression of NOD1 and NOD2 in CRC. NOD1 and NOD2 were both increased in the TCGA database, our patient cohort and additional databases in the Oncomine® Platform. NOD1 and NOD2 are PRRs, which recognize bacterial peptidoglycan, leading to the activation of NF-kB and MAPK pathways. NOD2 showed a strong genetic association with Crohns's disease, and NOD2 polymorphisms have been associated with gastric cancer and gastric lymphoma induced by *H. pylori* infection [20]. In a most simplistic case, increased NOD1 and NOD2 in CRC could result in increased NF-kB and MAPK signaling, thus enhancing tumorigenesis in CRC [21].

In summary, various NLR family members have been shown to influence murine models of CRC while this report analyzed the expression of multiple NLRs in human CRC. We have done extensive analyses that revealed the potential value of NLR and AIM2 genes as biomarkers of CRC and cancer progression. Reduced expression of these genes showed significant correlation with major clinical characteristics of CRC, and a subgroup of NLRs revealed tumor stage-specific reduced expression. Thus, NLRs potentially have diagnostic values as biomarkers and may represent promising targets for cancer therapy and prevention. Understanding the mechanisms underlying the function of these unique NLRs will assist in the design of future therapeutic strategies targeting a wide spectrum of inflammatory diseases and cancer. Beyond colon cancer, NLR inflammasome activation may also play important roles in other types of cancer, including breast cancer, skin cancers, and virus-associated hepatocellular carcinoma.

## MATERIALS AND METHODS

### Datasets and Oncomine^®^ Platform Bioinformatics

We used the gene search function of Oncomine^®^ Platform (www.oncomine.org) to locate microarray studies focusing on expression of NLRs and AIM2 in CRC. We used the default parameters on the site for NLRs and Aim2 expression analysis (threshold fold change greater or equal to 1.5X, p value greater or equal to IE-4 and gene rank in the top 10%). Data analysis was performed as fold change comparing normal tissue with CRC samples. Gene lists based on fold change were obtained from ten CRC datasets which were named by the convention based on the first author and numbers of patient (CRC/normal controls) as follows: Gaedcke [65/65] [36], Skrzypczak [81/24] [37], Hong [70/12] [38], TCGA colon cancer [215/22] [18], Kaiser [100/5] [39], Graudens [18/12] [40], Ki [50/28] [41], Gaspar [56/22][42], Notterman [18/18] [43] and Sabates-Bellver [32/32] [44]. Following the expression analysis of NLRs and AIM2 from those databases, results were sorted based on the *p* value and then the log­-transformed median centered raw data were downloaded from Oncomine® Platform. The boxplots and dot figures were created using GraphPad software.

### Clinical specimens

The IRB was approved by the Institutional Research Ethics Committees in Xijing Hospital, Xi'an, China. Samples that were used for our analyses were numbered and have not included any patients’ information that is protected under the IRB agreement. Bio specimens were collected from 40 patients who were newly diagnosed with colon or rectum adenocarcinoma that had undergone surgical resection during 2014 at the Xijing Hospital in Xi'an, China. Detailed information characterizing patients used in our studies are listed in Supplementary Table 1. All 40 colorectal cancer patients included in our studies were very carefully selected in the pre-screening process, where the H&E staining as shown in Supplementary Figure 6 was used to confirm the tumor formation in colorectal samples and normal crypt formation in healthy controls.

In addition every patient has been thoroughly interviewed by the doctors from the Department of Gastroenterology of Xijing Hospital; Xi'an in China and had no prior treatment or taken any medications prior the surgery. Violation any of those parameters were a major factor that would have excluded them from the studies. Immediately after the surgery, the excised colon tumor as well as the healthy colon control was sent to the Department of Pathology for pathological analysis and H&E staining (Supplementary Figure 6). Each Hematoxylin and Eosin staining (H&E) specimen was reviewed in China by board-certified pathologists (Dr. Peizhen Hu and Dr. Zengsan Li). Each adjacent normal tissue specimen was taken at the distance greater than 2cm from the actual tumor. Cases were staged according to the AJCC (American Joint Committee on Cancer staging system) [18]. Each tumor specimen weighed at least 60 mg and was typically under 200 mg. All of the specimens were formalin fixed and paraffin embedded (FFPE), histologic sections were obtained from top and bottom portions of each specimen. They confirmed that each tumor sample was histologically consistent with colon adenocarcinoma and that tumor cells were not contained in the adjacent normal specimen (Supplementary Figure 6).

### RNA extraction from FFPE tissue

RecoverAll^TM^ Total Nucleic Acid Isolation Kit was used according to the manufacturer's protocol to isolate RNA from FFPE Tissues (AM1975, Ambion, Austin, TX) with optimization in order to obtain high quality materials for analysis. Briefly, 1 ml of 100% xylene was added to 3 different pieces of 20 mm thick FFPE sections and samples were incubated for 3 min at 50°C to remove traces of paraffin, and then centrifuged for 2 min to discard the xylene. Pellets were washed twice with 1 ml 100% ethanol. Tissues were digested with 200μl digestion buffer with protease K at 50°C overnight and then treated with DNase I. After washing, total RNA was eluted with distilled water, and RNA concentration was measured using the Nanodrop 2000 spectrophotometer.

### Quantitative real-time PCR

IScript™ cDNA Synthesis Kit 170-8891(Bio-Rad Laboratories, Inc. CA) was used to make cDNA. Quantitative RT-PCR was performed on a ViiA™ 7 Real-Time PCR System. Quantitative PCR for human NLRP1 (Hs00248187_m1), NLRP3 (Hs00918082_m1), NLRP4 (Hs00370499_m1), NLRP6 (Hs00373246_m1), NLRP12 (Hs00536435_m1), NLRC3 (Hs01054716_m1), NLRC4 (Hs00368367_m1), NLRC5 (Hs00260008_m1), NLRX1 (Hs00226360_m1), NOD1 (Hs00196075_m1), NOD2 (Hs00223394_m1) and AIM2 (Hs00223394_m1) was performed by using TaqMan primer/probe sets and master mix (Applied Biosystems). To normalize the qPCR results, GAPDH (Hs02758991_g1), ACTB (Hs01060665_g1) and RNA18S (Hs03928985_g1) were chosen as reference genes. All samples were loaded in triplicates. Relative mRNA expression levels were compared via the 2^−ΔΔCT^ methods or log-transformed [45].

### Statistical analysis

Data are presented as mean ± SEM. Ratios of real-time PCR expression values of case matched normal and cancer tissues were used after normalization by the mean expression value of internal control genes: ACTB, GAPDH and 18S RNA. Differences between two sample groups were analyzed using the paired or unpaired Student's t-test function of GraphPad Prism version 4 software (Mann-Whitney unpaired test or Wilcoxon matched-pairs signed test). The association between gene expression levels and clinical parameters was addressed by analysis of variance (ANOVA). *p* < 0.05 was considered statistically significant. The Spearman correlation and linear regression analyses were performed using GraphPad. Interpretation of r value was conducted based on the following criteria: values greater than 0 and up to 1 indicated that two variables were changing in the same direction in a correlative fashion, while values below 0 down and up to −1 indicated two variables that were changing in the opposite direction.

## SUPPLEMENTARY MATERIAL FIGURES AND TABLE


