# Supplementary material for: Expression profile of innate immune receptors, NLRs and AIM2, in human colorectal cancer: correlation with cancer stages and inflammasome components
Source: Oncotarget. 2015 Sep 10;6(32):33456–69. doi: 10.18632/oncotarget.5587 (PMC4741778; doi:10.18632/oncotarget.5587)
Supplement: Supplementary file 1 [file oncotarget-06-33456-s001.pdf]

Expression profile of innate immune receptors, NLRs and AIM2, in human colorectal cancer: correlation with cancer stages and inflammasome components

Supplementary Material

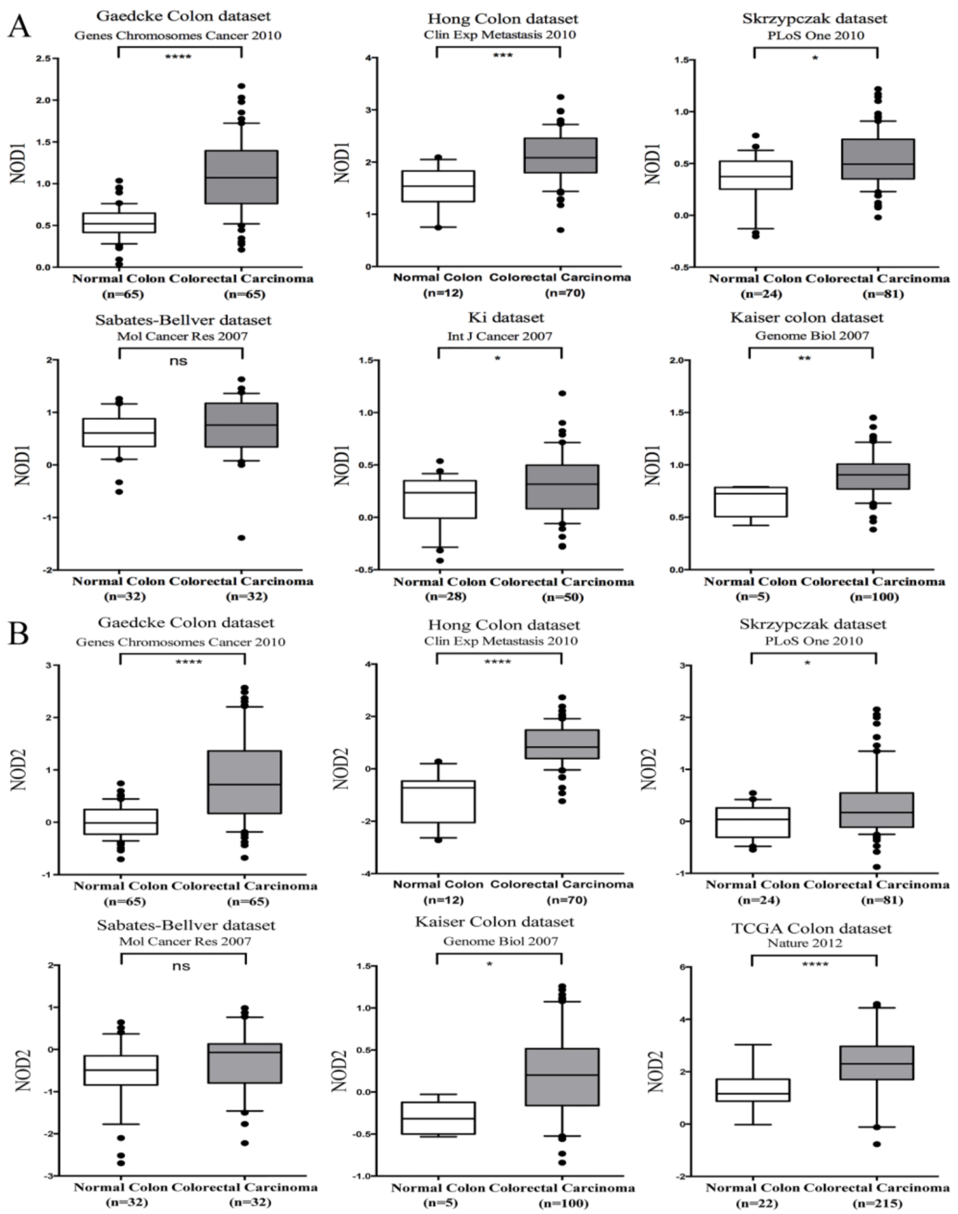

Supplementary Figure 1. Analysis of microarray expression data for NOD1 and NOD2 levels from the various Oncomine® Platform databases. Log2 median-centered ratio expression is present for 6 different datasets. * P< 0.05; ** P< 0.01; ** P< 0.001**** P< 0.0001.

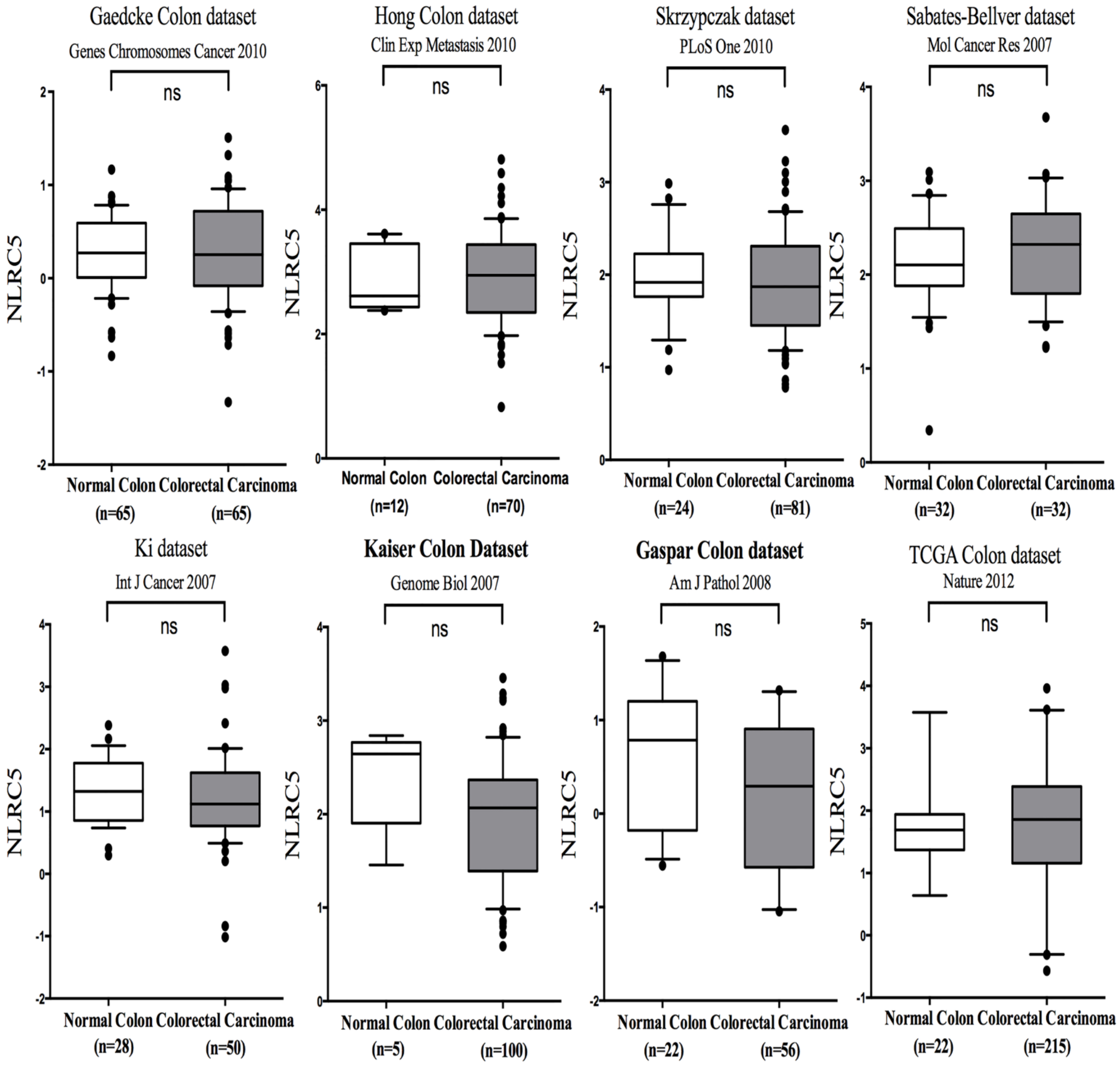

Supplementary Figure 2. Analysis of microarray expression data for NLRC5 levels from the various Oncomine® Platform databases. Log2 median-centered ratio expression is present for 8 different datasets. * P< 0.05; ** P< 0.01; ** P< 0.001**** P< 0.0001.

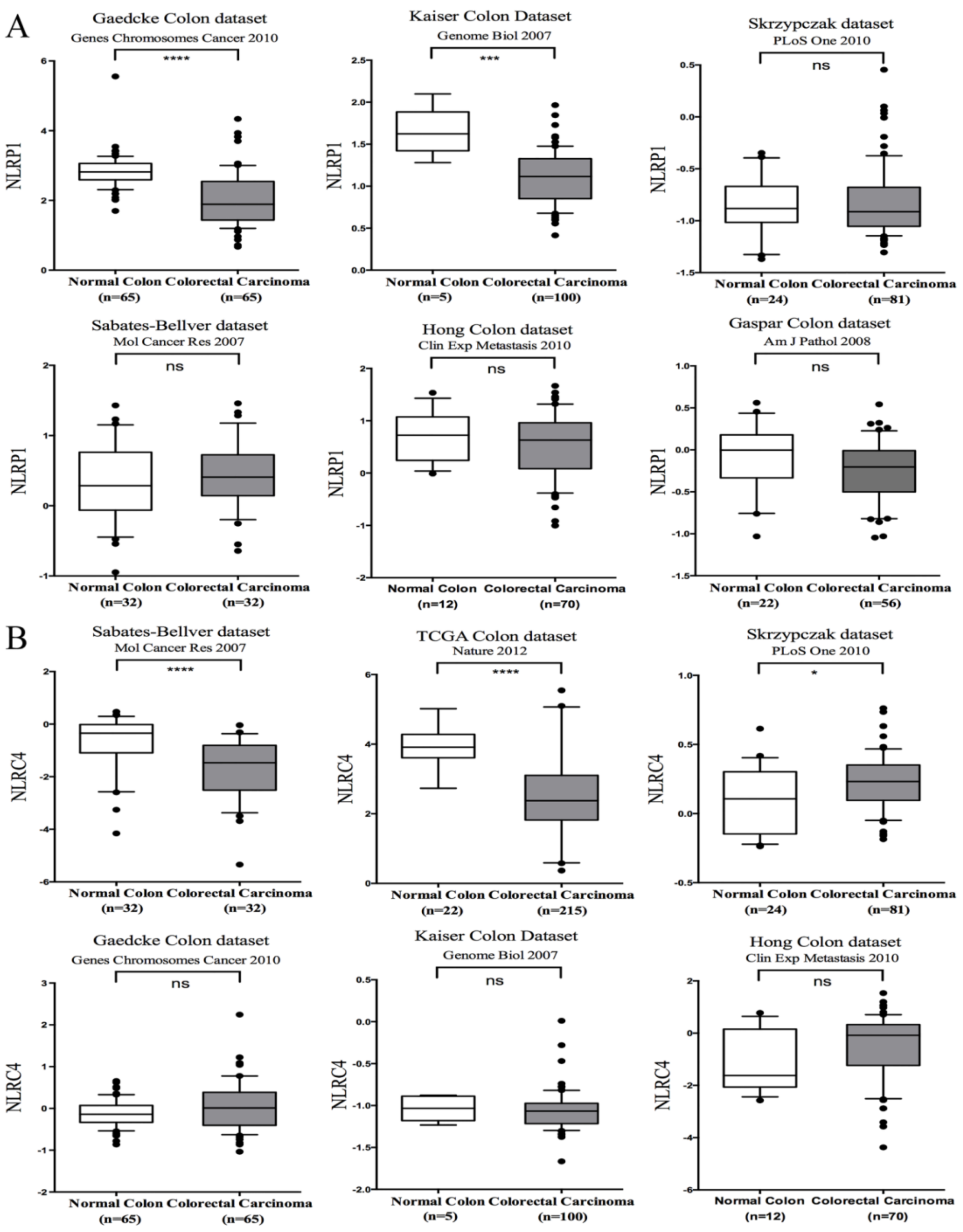

Supplementary Figure 3. Analysis of microarray expression data for NLRP1 and NLRC4 levels from the various Oncomine® Platform databases. Log2 median-centered ratio expression is present for 6 different datasets. * P< 0.05; ** P< 0.01; ** P< 0.001**** P< 0.0001, ns: no statistical difference.

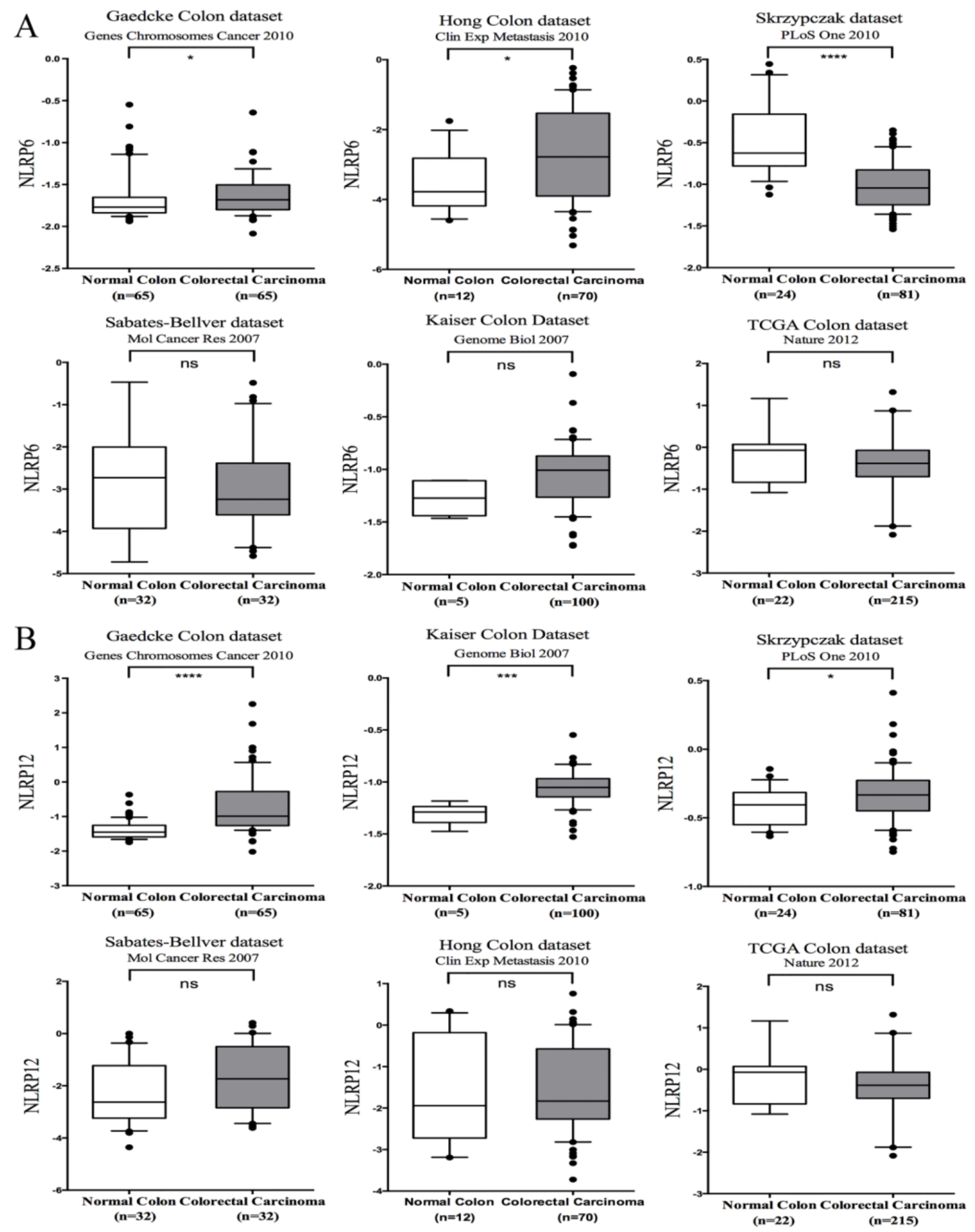

Supplementary Figure 4. Analysis of microarray expression data for NLRP6 and NLRP12 levels from the various Oncomine® Platform databases. Log2 median-centered ratio expression is present for 6 different datasets. * P< 0.05; ** P< 0.01; ** P< 0.001**** P< 0.0001, ns: no statistical difference.

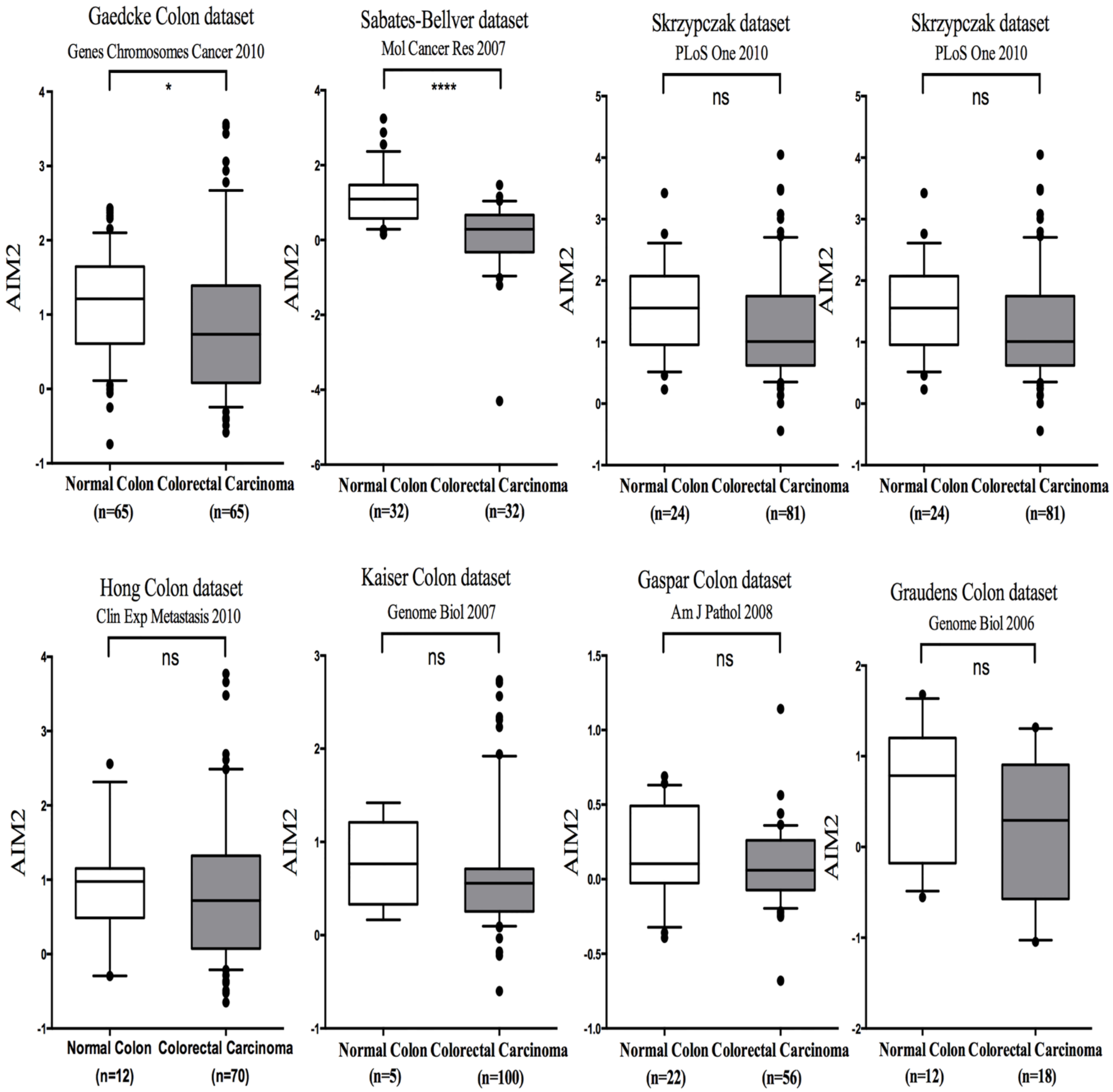

Supplementary Figure 5. Analysis of microarray expression data for AIM2 levels from the various Oncomine® Platform databases. Log2 median-centered ratio expression is present for 8 different datasets. * P< 0.05; ** P< 0.01; ** P< 0.001**** P< 0.0001. ns: no statistical difference

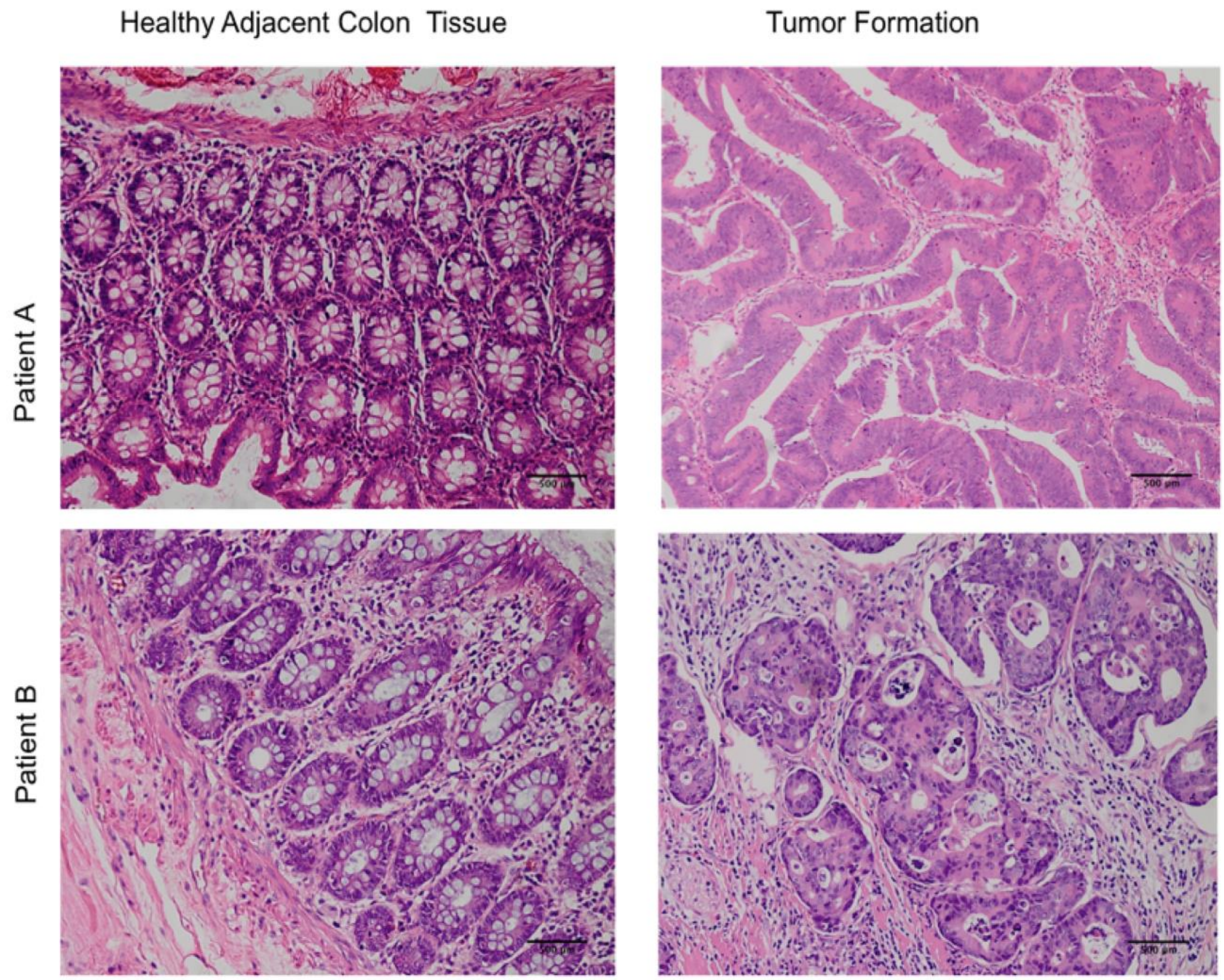

Supplementary Figure 6. H&E representation of the colon cancer and healthy adjacent tissues, where you can clearly distinguish healthy tissues in adjacent healthy controls versus the adenomas isolated from colon tumors.

Supplementary Table 1. Detailed patient information for all 40 clinical colorectal cancer samples that were utilized in experimental analyses.

	**Patient**	**Sex**	**Age**	**Location**	**Grade**	**pT UICC**	**pN UICC**	**pM UICC**	**AJTT STAGE**	**Dukes**
**1**	2260	F	58	Cecum	M	T3	N0	M0	IIA	B
**2**	2261	F	52	sigmoid colon	P	T3	N1	M1	IV	D
**3**	2262	F	65	sigmoid colon	M	T3	N2	M1	IV	D
**4**	2403	F	50	sigmoid colon	M	T3	N0	M0	IIA	B
**5**	2460	F	57	sacending colon	G	T1	N0	M0	I	A
**6**	2461	F	43	sigmoid colon	P	T3	N2	M1	IV	D
**7**	2462	F	68	sacending colon	P	T4	N0	M1	IV	D
**8**	2531	F	69	sigmoid colon	M	T3	N2	M1	IV	D
**9**	2533	M	37	transverse colon	M	T3	N1	M0	IIIB	C
**10**	2593	M	38	transverse colon	P	T3	N2	M1	IV	D
**11**	2647	M	28	sacending colon	M	T3	N0	M0	IIA	B
**12**	2648	M	80	transverse colon	M	T4	N2	M1	IV	D
**13**	2746	F	72	sigmoid colon	M	T3	N0	M0	IIA	B
**14**	2747	F	49	rectum	G	T4	N0	M0	IIB	B
**15**	2748	F	59	sigmoid colon	M	T4	N0	M0	IIB	B
**16**	2829	F	59	sigmoid colon	P	T3	N1	M0	IIIB	C
**17**	2830	M	72	sacending colon	G	T3	N0	M0	IIA	B
**18**	2833	M	81	Cecum	M	T2	N0	M0	I	A
**19**	2883	M	40	sigmoid colon	P	T3	N2	M1	IV	D
**20**	2884	M	46	sigmoid colon	M	T3	N2	M1	IV	D
**21**	2945	F	40	sacending colon	M	T3	N0	M0	IIA	B
**22**	3002	F	42	sigmoid colon	G	T4	N1	M1	IV	D
**23**	3061	F	80	sigmoid colon	P	T3	N1	M1	IV	D
**24**	3243	F	59	sacending colon	P	T4	N0	M0	IIB	B

**25**	3244	F	80	sacending colon	P	T3	N0	M0	IIA	B
**26**	3293	M	75	descending colon	M	T4	N0	M0	IIB	B
**27**	3300	F	66	sigmoid colon	G	T3	N1	M1	IV	D
**28**	3431	F	65	sacending colon	M	T3	N2	M1	IV	D
**29**	3433	F	54	sigmoid colon	M	T3	N0	M0	IIA	B
**30**	3434	M	65	sigmoid colon	M	T3	N2	M1	IV	D
**31**	3484	M	63	transverse colon	M	T3	N1	M1	IV	D
**32**	3485	F	76	descending colon	G	T3	N0	M0	IIA	B
**33**	3486	F	77	transverse colon	P	T3	N0	M0	IIA	B
**34**	3664	M	58	sacending colon	G	T4	N0	M0	IIB	B
**35**	3710	F	65	sigmoid colon	M	T4	N1	M1	IV	D
**36**	3712	M	80	sigmoid colon	P	T4	N0	M0	IIB	B
**37**	3763	F	42	transverse colon	G	T3	N0	M0	IIA	B
**38**	3768	F	68	sacending colon	M	T3	N0	M0	IIA	B
**39**	3854	F	60	transverse colon	M	T3	N1	M1	IV	D
**40**	4002	F	51	Cecum	P	T2	N0	M0	I	A
